# Shearing Tooth Morphology May Allow Sharks to Access Higher Trophic Levels at Smaller Sizes

**DOI:** 10.1002/ece3.71722

**Published:** 2025-07-29

**Authors:** Sabrina Riverón, Vincent Raoult, David J. Slip, Federico Mas, Martín Laporta, Inés Pereyra, Santiago Silveira, Robert G. Harcourt

**Affiliations:** ^1^ Marine Predator Research Group, School of Natural Sciences Macquarie University North Ryde New South Wales Australia; ^2^ Dirección Nacional de Recursos Acuáticos (DINARA) Montevideo Uruguay; ^3^ School of Environment and Science Griffith University Gold Coast Queensland Australia; ^4^ School of Environmental and Life Sciences University of Newcastle Ourimbah New South Wales Australia; ^5^ Marine Ecology Group, School of Natural Sciences Macquarie University North Ryde New South Wales Australia; ^6^ Taronga Institute of Science and Learning Taronga Conservation Society Australia Mosman New South Wales Australia; ^7^ Unidad de Gestión Pesquera Atlántica Dirección Nacional de Recursos Acuáticos (DINARA) La Paloma Uruguay; ^8^ Campus DoMar, International Campus of Excellence University of Santiago de Compostela A Coruña Spain

**Keywords:** *Carcharias taurus*, *Notorynchus cepedianus*, prey choice, southwestern Atlantic Ocean, stable isotopes, stomach content, teeth, trophic ecology

## Abstract

The availability of prey in an environment does not ensure that a predator will consume it: prey must also be detected, captured, and successfully handled. The morphology of the predator and prey imposes limitations on prey selection due to biomechanical constraints, making some prey functionally inaccessible. Morphological factors, including but not limited to tooth shape, body size, and mouth gape, therefore impose constraints on predator trophic niches. We assessed how two important components of trophic morphology (tooth shape and body length) may influence prey selectivity and trophic niche in two large‐bodied sympatric sharks with contrasting foraging strategies. The first species captures prey using spear‐shaped, grasping teeth (grey nurse/sand tiger/raggedtooth shark, *Carcharias taurus* ), while the second has multi‐cuspid cutting teeth used to serrate larger prey (sevengill shark, *Notorynchus cepedianus* ). Stomach content analysis and isotopic values of δ^13^C, δ^15^N, and δ^34^S from muscle and liver were used to characterize isotopic niche and prey selection. As gape‐limited grey nurse sharks grew, their consumption of teleosts decreased inversely to chondrichthyans. By contrast, non‐gape limited sevengill sharks consumed teleosts and chondrichthyans in similar proportions, along with marine mammals, but with no clear relationship to body size. As body length increased, both species consumed prey from higher trophic levels (higher δ^15^N values), but sevengill sharks accessed prey at relatively higher trophic levels. Values of δ^13^C and δ^34^S remained relatively unchanged with body length presumably because mouth gape and dentition do not limit access to pelagic or benthic food webs. Although many other morphological factors, such as swim performance, biomechanics, or behavior, could also drive the results we observed, it is clear that morphological characteristics play an important role in prey selection and may be the primary mechanism facilitating resource partitioning in large sympatric predators. Their inclusion in ecological studies will help understand prey choice and how it shapes trophodynamics in marine ecosystems.

## Graphical abstract

Predator morphology imposes limitations on prey selection due to biomechanical constraints, making some prey functionally inaccessible and thereby constraining predator trophic niches. We assessed how two key components of trophic morphology—tooth shape and body size—affect prey selectivity and trophic niche in two sympatric sharks with contrasting foraging strategies: the gape‐limited grey nurse shark ( *Carcharias taurus* ) and the non‐gape‐limited sevengill shark ( *Notorynchus cepedianus* ). Our findings demonstrate that predator morphology plays a significant role in prey selection and may be the primary mechanism facilitating resource partitioning in sympatric species and influencing trophodynamics in marine ecosystems.

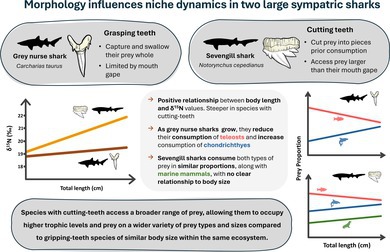

## RESUMEN

La disponibilidad de presas en un ambiente no garantiza su consumo por parte de un depredador; la presa también debe ser detectada, capturada y manipulada con éxito. La morfología tanto del depredador como de la presa impone restricciones biomecánicas que pueden hacer que algunas presas sean funcionalmente inaccesibles y, por tanto, afectar la selección de presas. Factores morfológicos, tales como la forma de los dientes, el tamaño corporal, la apertura bucal, entre otros, imponen restricciones a los nichos tróficos de los depredadores. En este estudio se evaluó cómo dos importantes componentes de la morfología trófica (forma de los dientes y longitud corporal) pueden influir en la selectividad de presas y en el nicho trófico de dos especies simpátricas de tiburones de gran tamaño, con estrategias alimentarias contrastantes. La primera especie, el tiburón sarda ( *Carcharias taurus* ), presenta dientes largos, estrechos y puntiagudos, adaptados para capturar y tragar presas enteras; mientras que la segunda, el tiburón pintarroja ( *Notorynchus cepedianus* ), posee dientes multicúspides cortantes, especializados en despedazar presas de mayor tamaño. Se utilizaron análisis de contenido estomacal y valores isotópicos de δ^13^C, δ^15^N y δ^34^S en músculo e hígado para caracterizar el nicho trófico y la selección de presas en ambas especies. Conforme el tiburón sarda crece, limitado por el tamaño de su apertura bucal, su consumo de teleósteos disminuye en proporción inversa al de condrictios. En contraste, el tiburón pintarroja, al no tener limitaciones asociadas al tamaño de su abertura bucal, consume teleósteos y condrictios en proporciones similares, además de mamíferos marinos, aunque sin una relación clara con su tamaño corporal. En ambas especies, el aumento de la longitud corporal se asocia con el consumo de presas de niveles tróficos más altos (reflejado en mayores valores de δ^15^N), aunque el tiburón pintarroja accede a presas de niveles tróficos relativamente superiores. Los valores de δ^13^C y δ^34^S se mantienen relativamente invariables con la longitud corporal, presumiblemente porque el tamaño de la apertura bucal y la dentición no limitan el acceso a redes alimenticias pelágicas o bentónicas. Si bien otros factores morfológicos, como aquellos que afectan el rendimiento del nado, la biomecánica o el comportamiento, también podrían influir en los resultados observados, está claro que ciertas características morfológicas desempeñan un papel importante en la selección de presas y pueden constituir el principal mecanismo que facilita la repartición de recursos en grandes predadores simpátricos. La inclusión de estos caracteres en estudios ecológicos ayuda a comprender la selección de presas y cómo ello moldea la trofodinámica en los ecosistemas marinos.

## 1 Introduction

The feeding ecology of marine predator populations, and therefore their trophic niche, is defined by a combination of biotic and abiotic factors. Biotic factors refer to all living organisms in an ecosystem, which together constitute the food web. Abiotic factors, such as temperature and salinity, determine in part the presence, distribution, and abundance of prey and their consumers within an ecosystem (Castillo et al. 1996; Harrison and Whitfield 2006; Bailleul et al. 2007; Domenici et al. 2007; Smyth and Elliott 2016; Niella et al. 2022). Together, biotic and abiotic components of an ecosystem are drivers of inter‐and intraspecific competition for resources (Chase et al. 2002; Ward et al. 2006; Svanbäck and Bolnick 2007). A decrease in prey abundance may lead to increased competition among its consumers; conversely, if the resource is more abundant, competition may relax. However, the presence of potential prey in the environment does not imply that a predator will invariably feed upon that prey. Predators have intrinsic factors that enhance or constrain their feeding abilities, including morphological, physiological, and behavioral features (Horn and Ferry‐Graham 2006). Morphological characteristics constitute one of the final modes of prey selection and determine whether a predator is capable of consuming prey once all other requirements, such as prey availability, have been met. Accordingly, these morphological characteristics exert differential constraints on marine predators with different morphological features (Ferry 2015; Bazzi et al. 2021).

Feeding phenotypes can have different evolutionary pathways, with distinct processes or routes through which species evolve over time. These trophic phenotypes originate from adaptive radiations that begin with habitat differentiation and continue with the evolution of divergent feeding morphological structures (Streelman and Danley 2003; Grossnickle 2020; Bazzi et al. 2021; Gayford and Jambura 2025). Optimal foraging theory proposes that changes in the trophic morphology (morphological characters associated with the feeding ecology of individuals) will maximize the energy gain from feeding on a preferred available prey (MacArthur and Pianka 1966; Pyke 1984; Winkler et al. 2017). However, despite its fundamental role in feeding ecology, morphological traits other than total size are rarely considered as parameters in trophic models (Ferry‐Graham et al. 2002), even though this mechanism may explain the ecological patterns obtained through other analytical approaches (e.g., stomach content and/or stable isotope analysis) (Verde Arregoitia et al. 2017; Keppeler et al. 2020). This is concerning because, while many feeding‐associated structures are closely associated with size, many are not, and they show positive or negative allometric relationships with size (e.g., Ahnelt et al. 2020; Gayford, Whitehead, et al. 2023) or are constrained by other biological requirements (e.g., respiration, Prinzing et al. 2023).

Morphological structures directly associated with feeding are key to determining which available prey items will ultimately become part of an animal's diet (Horn and Ferry‐Graham 2006). A strong relationship between diet and feeding morphology has been reported for many taxonomic groups, including terrestrial vertebrates (Grossnickle 2020; Figueira et al. 2023), sharks (Scacco et al. 2010; Bazzi et al. 2021; López‐Romero et al. 2023), bony fishes (Ferry‐Graham et al. 2001; Olivier et al. 2019), marine turtles (Figgener et al. 2019), and invertebrates (DeVries 2017). Morphological structures are a strong proxy to infer dietary habits such that it is even possible to infer the dietary habits of extinct species based on characteristics of living equivalents, for example, shark (Bazzi et al. 2021) and mammalian (Croft et al. 2011; Verde Arregoitia et al. 2017) teeth used as references for the fossil record. Given that teeth are often the only fossilized remains available for some animals, particularly sharks, tooth morphology plays a critical role in elucidating the ecology of extinct species (e.g., Bazzi et al. 2021; Cooper et al. 2023).

Individuals seek to maximize their net energy gain from predation, and this involves a combination of time and energy loss due to the predation event, which includes searching, handling, processing, and digestion (Willson and Hopkins 2011; Hocking et al. 2021). In predators, increased body size generally improves the ability to handle larger prey and therefore access a new range of prey items (Wainwright and Richard 1995; MacNulty et al. 2009; Cuthbert et al. 2020). Variation in diet associated with changes in predator body size occurs throughout ontogeny, either as a direct consequence of increased body size or due to changes in their feeding structures (Stoner and Livingston 1984; Ward‐Campbell and Beamish 2005; Powter et al. 2010; Ferrara et al. 2011; Davis et al. 2012; Türtscher et al. 2022). Additionally, ontogenetic changes in fin shape impact swimming efficiency in sharks and have been associated with ontogenetic shifts in diet (e.g., Ahnelt et al. 2020; Gayford, Whitehead, et al. 2023; Gayford, Godfrey, and Whitehead 2023; Gayford, Whitehead, and Jaquemet 2024; Gayford, Waghe, et al. 2024; Irschick and Hammerschlag 2015; Seamone et al. 2024). However, while these structures may be associated with ontogenetic changes in diet, mouth‐associated feeding structures may have more pronounced effects on the ability of sharks to consume prey, as they do in bony fishes (Bonato et al. 2017; Winkler et al. 2017).

For most marine predators, optimization of the feeding process is explicitly related to the mouth and associated structures. Mouth gape is closely associated with the maximum prey size that a predator can consume, especially those that swallow their prey whole (Karpouzi and Stergiou 2003; Mihalitsis and Bellwood 2017). Species with teeth that can shear their prey into smaller pieces can target individuals larger than their gape size, freeing them from these constraints and providing them with a greater range of prey sources (Lucifora et al. 2005; Ferrara et al. 2011). Among dietary structures, tooth morphology has been the most correlated with the feeding habits of predators (Wilga and Ferry 2015; Van Valkenburgh 2019). Consequently, we expect that differences in predator body size and tooth shape will have a strong relationship with their trophic niche by constraining access to different types of prey.

**FIGURE 1:**
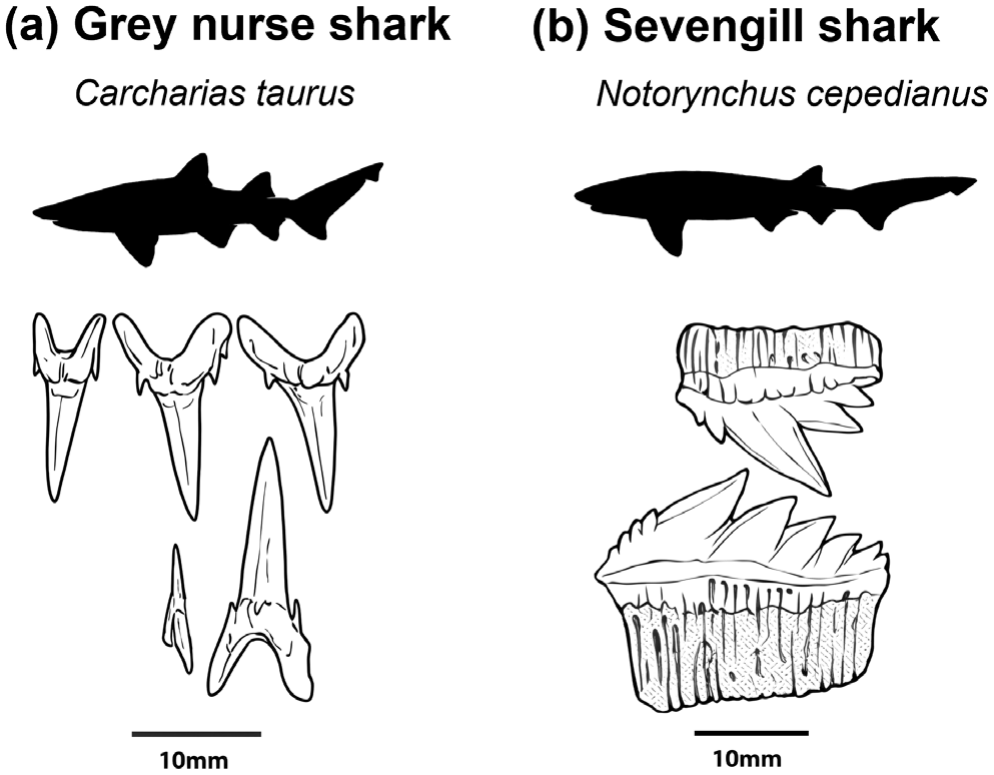
Tooth morphology of grey nurse sharks (a, *Carcharias taurus* ) and sevengill sharks (b, *Notorynchus cepedianus* ). Modified from Ebert et al. (2021).

Sevengill sharks ( *Notorynchus cepedianus* ) and grey nurse sharks ( *Carcharias taurus* ) are two broadly distributed coastal shark species whose sympatry has been documented in the Southwest Atlantic, particularly along the coasts of Uruguay and central‐northern Patagonia (Lucifora et al. 2002; De Wysiecki et al. 2025). Despite their similar body sizes (85–325 cm grey nurse sharks and 35–295 cm sevengill sharks), they exhibit contrasting trophic morphology (Figure 1) (Ebert et al. 2021). These two sharks are from orders that diverged nearly 200 mya (Carcharhiniforms and Hexanchiformes; see Klug and Kriwet 2013), and as a result, their coexistence offers an interesting case study where two similar‐sized predators have evolved in an ecosystem where they have access to similar prey. Sevengill sharks have a single characteristically posteriorly placed dorsal fin, whereas grey nurse sharks have more widespread medial dorsal fin placement (Ebert et al. 2021). Grey nurse sharks have spear‐shaped teeth adapted to capture and swallow their prey whole with minimal handling (Lucifora et al. 2001; Ferrara et al. 2011; Lucifora, García, and Escalante 2009), limiting their ability to consume prey larger than their mouth gape and therefore restricting the trophic levels over which they can feed; this means the maximum size of prey they can consume is closely related to their total length (Smale 2005). In contrast, sevengill sharks have broad‐based multi‐cusped cutting teeth which they use to cut their prey into pieces prior to consumption (Ebert 1991), allowing them to access prey much larger than their mouth gape from higher trophic levels (e.g., marine mammals) (Crespi‐Abril et al. 2003; Lucifora et al. 2005). Grey nurse and sevengill sharks are sympatric but have distinct morphologies; these two species can be used to assess how morphological factors such as dentition may help to determine the trophic niche.

We hypothesize that, despite having otherwise similar total lengths, cutting‐toothed sevengill sharks will have access to a broader range of prey sizes than the grasping grey nurse sharks, and this will be reflected in a broader trophic niche and access to prey over higher trophic levels. Thus, this study assesses how two important components of trophic morphology (tooth shape and body size) may influence prey selectivity and trophic niche in two large‐bodied sympatric predators with contrasting foraging strategies.

## 2 Methods

### 2.1 Study Site and Sampling

**FIGURE 2:**
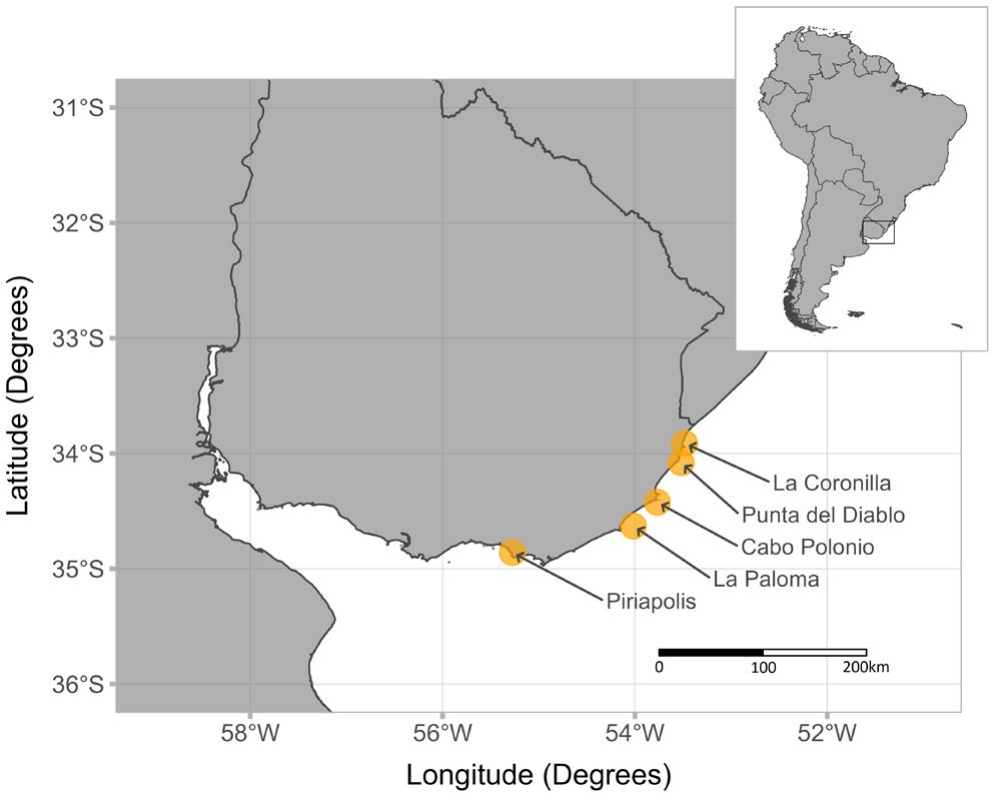
Localities along the Atlantic coast of Uruguay (yellow circles) where samples were obtained from artisanal and recreational fisheries.

Samples of muscle, liver, and whole stomachs from dead grey nurse and sevengill sharks were obtained from artisanal and recreational fisheries located along the Atlantic coast of Uruguay (Figure 2). Sharks were primarily caught within the coastal zone, from the shoreline to 10 nautical miles (nm) offshore, using gillnets (artisanal fisheries) or rod and reel (recreational angling). Samples were collected over two consecutive shark fishing seasons between spring (September to December) and summer (December to February) of 2018–2019 and 2019–2020 seasons when both species co‐occur in the same areas and have access to the same food resources (Praderi 1985; Silveira et al. 2018). Fine‐scale habitat use and movements for these species remain poorly understood in the southwest Atlantic, with data being limited or entirely lacking in certain areas. In Uruguay and Argentina, it is common for individuals of both species to be caught in the same fishing haul (Lucifora et al. 2002; Silveira et al. 2018). However, it is also true that some fishers typically use different fishing zones for each species, depending on the areas where their individuals aggregate (rocky bottom for grey nurse sharks, and sandy/muddy bottoms for seven‐gill sharks). Within the first hour after landing, individuals were identified, sexed, and measured for the total length (TL, *sensu* Ebert et al. 2021). Liver was sampled from the posterior portion of either lobe, while white muscle was sampled from the ventral region at the base of the pelvic fins. Whenever possible, the stomach was removed and sealed with zip ties at the oesophagus and at the anterior end of the spiral valve. Samples were stored at −20°C until they were processed in the laboratory. Samples were collected in collaboration with local fishers as part of the participatory fisheries monitoring programs conducted by the Atlantic Fisheries Management Unit of the National Directorate of Aquatic Resources (UGEPA‐DINARA, Ministry of Livestock, Agriculture and Fisheries) (Laporta et al. 2018; ICES 2020), under permit 252/2018 issued by DINARA.

### 2.2 Stable Isotope Analysis

We characterised consumers' isotopic niches and estimated their trophic role using isotopic values of δ^13^C, δ^34^S, and δ^15^N. Stable isotopes of carbon (δ^13^C) and sulphur (δ^34^S) indicate resource use by consumers (e.g., coastal vs. oceanic, benthic vs. pelagic), while the relative trophic position is indicated by nitrogen isotopic values (δ^15^N) (Post 2002). Stability over time in prey choice was assessed by analysing isotopic values in the liver and muscle, which represent tissues with short‐ and long‐term dietary incorporation rates, respectively (Hussey et al. 2012).

Muscle and liver samples of grey nurse (*n* = 104) and sevengill sharks (muscle *n* = 34, liver *n* = 31) were cleaned with de‐ionised water to avoid any residue that could affect the isotopic signals. The sex distribution was as follows: for grey nurse sharks, 44 females, 48 males, and one individual of unknown sex; for sevengill sharks, muscle samples included 21 females, 11 males, and two of unknown sex, while liver samples included 20 females, 10 males, and one of unknown sex. Samples from the 2018 to 2019 fishing season were freeze‐dried, while samples from 2019 to 2020 were oven‐dried at 60°C for 72 h. The method of drying does not affect isotopic values (Akamatsu et al. 2016; Bashir et al. 2020). Dried samples were ground into a fine powder and homogenised using an IKA A11 Basic Analytical Mill (IKA‐Werke GmbH & Co. KG, Staufen, Germany).

Lipid extraction was conducted on approximately 1 g of tissue using a 2:1 chloroform‐methanol solution adapted from Folch et al. (1957). The process was repeated until the supernatant was clear, ensuring lipids were removed. To eliminate the remaining solvent, samples were dried at room temperature for 48 h or until the solvent completely evaporated.

Shark tissues are rich in urea and trimethylamine N‐oxide (TMAO), which affects the δ^15^N and δ^13^C isotopic values, leading to biases in ecological interpretations (Kim and Koch 2012; Li et al. 2016; Bennett‐Williams et al. 2022). Therefore, we performed lipid extraction, followed by urea extraction, using an adaptation of the Kim and Koch (2012) protocol. Samples were rinsed with 5 mL of deionised water for 10 min, vortexed for 1 min, then centrifuged, and the supernatant was discarded. This procedure was repeated three times. Samples were dried for 24 h in a drying oven at 60°C or until the sample was dried entirely (usually no more than 48 h). Dried samples were then weighed in tin capsules and sent for analysis.

Stable isotope values of δ^13^C, δ^15^N, and δ^34^S were analyzed at the Stable Isotope Laboratory at the University of Hong Kong, using a continuous flow‐isotope ratio mass spectrometer Thermo Scientific EA IsoLink IRMS System (Thermo Fisher Scientific Inc., Massachusetts, USA). The following international standards were used for data normalization: USGS‐40 and USGS‐41a for ^15^N and ^13^C; IAEA‐S‐1 and IAEA‐S‐2 for ^34^S. These international standards were calibrated to Vienna Pee Dee Belemnite (VPDB) for δ13C values, atmospheric N for δ15N values, and Vienna Cañon Diablo troilite (VCDT) for δ34S values. Analytical accuracy was evaluated using the reference material USGS‐42 (δ^15^N = 0.1–0.3, δ^13^C = 0.0–0.1 and δ^34^S = 0.1–1.0).

### 2.3 Stomach Content Analysis

To test our hypothesis about differences in diet breadth, we quantified the diet of each species using stomach content analysis. Stomach contents were rinsed with fresh water, and items were sorted using a series of 4, 2, and 1 mm metal mesh sieves. Prey items were identified to the lowest possible taxonomic group. Hard parts (e.g., otoliths, pharyngeal teeth plates, and other bones) were dried for identification. Otoliths were photographed with a stereomicroscope Nikon SMZ‐745T (Nikon Instruments Inc., Melville, USA) for identification using regional otolith guides as a reference (Volpedo and Echeverría 2000; Rossi‐Wongtschowski et al. 2014; Giaretta et al. 2017; Volpedo et al. 2017). Fish remains in advanced stages of digestion were identified based on extracted otoliths or skeletal morphology (axial, cranial, and appendicular). Bones were identified using the reference literature of the prey species (Dyer 2006; Deli Antoni et al. 2008; Tombari et al. 2010; Rodrigues and Bemvenuti 2011; Marceniuk et al. 2012; Perez Comesaña et al. 2014; Bemis et al. 2019; Colautti et al. 2020). Items identified as parasites (e.g., isopods, nematodes), secondary prey items (e.g., small bivalves), and other non‐dietary items (e.g., fishing gear fragments, sand) were excluded from the analysis. Secondary prey items were identified based on their size and ecological relevance to the studied species, in accordance with its known diet, size, feeding behaviour, and ecological niche. These items are likely to have been ingested incidentally by consuming a primary prey.

As sharks digest prey relatively quickly, we used a combination of a presence‐absence method (Frequency of occurrence, %*F*) and a numerical method (%*N*) (Hynes 1950; Hyslop 1980; Amundsen and Sánchez‐Hernández 2019) to describe the dietary composition of each species. The frequency of occurrence is defined as the number of stomachs in which a specific prey type was present and expressed as a percentage of the total number of stomachs with any prey type (Hyslop 1980). We calculated %*N* as the total number of individuals of a particular prey type as a proportion of the total number of prey items in all stomachs (Hynes 1950). Only stomachs with the prey content were considered for calculations of %*F* and %*N*.

### 2.4 Statistical Analyses

For each shark species, we used a cumulative prey curve to determine if the number of stomachs analysed was enough to accurately describe the diet of a particular predator (Ferry and Cailliet 1996; Cortés 1997). Cumulative prey curves were constructed using the software *EstimateS* version 9.1.0 (Colwell et al. 2004). The order in which the stomachs were analysed was randomised 100 times, and the mean cumulative number of new prey items was plotted against the number of stomachs examined. Individuals with empty stomachs and unidentified prey items were excluded from the calculations, resulting in 92 stomachs analysed for grey nurse sharks and 21 for sevengill sharks. The sample size was considered sufficient to describe diet if the curve reached an asymptote (Magurran 2004).

To assess whether the consumption of different prey types varies with body size, we analyzed the variation in the proportion of prey consumed and the isotopic values of δ^13^C, δ^15^N, and δ^34^S with total length. For the first analysis, we defined the proportion of prey consumed as the frequency of each prey type found in one stomach, using three types of prey: actinopterygii (bony fishes), chondrichthyes, and marine mammals. A generalized linear model was fitted for each species of shark. As the values for the proportion of prey are constrained between 0 and 1, we adjusted a function with a binomial distribution, which used a default logit link function. We used the proportion of prey consumed as the response variable and total length as the explanatory variable, with the type of prey included in the model as an interaction. Marine mammals were not included in the grey nurse shark model, as none were recorded as prey.

For the second analysis, we fitted linear models independently for each stable isotope and tissue type, using shark total length as a predictor variable. When a linear regression was fitted for the same tissue type and stable isotope for both species, the slopes were compared using a *t*‐test. We ran a linear regression between the stable isotope values and total length by tissue type, with species included as interactions. Since maternal influence can bias the isotopic values of neonate individuals in some shark species (Matich et al. 2010; Olin et al. 2011; Niella et al. 2021), individuals smaller or equal to the maximum reported birth size (110 cm TL for grey nurse sharks, Gilmore et al. 1983; 53 cm TL for sevengill sharks, Compagno 1984) were removed from the analyses.

Different tissue types have different turnover rates and can be used to look at different time‐spans, but these tissues often have different tissue‐specific isotopic enrichment factors (Hussey et al. 2010; Kim et al. 2012). To make liver isotopic values of δ^13^C and δ^15^N directly comparable to muscle and assess patterns over the short and long terms, we corrected liver values using diet‐tissue discrimination factors for lipid‐extracted samples published by Hussey et al. (2010). We used species‐specific values for grey nurse sharks, and for sevengill, we used the generalised estimate values for ‘*all sharks*’ (Hussey et al. 2010). The values of δ^15^N and δ^13^C of grey nurse shark liver were increased by 0.69‰ and 0.17‰, respectively. For sevengill sharks, liver δ^15^N and δ^13^C values were increased by 0.79‰ and 0.68‰, respectively. These values correspond to the differences between mean values of lipid‐extracted muscle samples and mean values for lipid‐ extracted liver published by Hussey et al. (2010). No correction was performed to δ^34^S values, as meta‐analysis suggests that this value is often not significantly different to zero for this isotope (Raoult et al. 2024).

Since sexual dimorphism in shark traits such as head shape and dentition has been previously documented (Powter et al. 2010; Berio et al. 2020), we tested whether isotopic values and the proportion of prey consumed differ between sexes across ontogeny. To do this, we ran linear models for each stable isotope using TL as a predictor variable and included sex as an interaction term. For the proportion of prey consumed (Actinopterygii and Chondrichthyes), we fitted a generalized linear model with a binomial distribution (logit link), using TL as the dependent variable and including sex as an interaction term. Due to the low number of sevengill sharks that consumed marine mammals (*n* = 3), it was not possible to test differences between sexes for this type of prey.

Linear models were fitted in R software version 4.4.2 (R Core Team 2024) using the functions ‘*lm*’ and ‘*glm*’ from the package ‘*stats*’. Assumptions of the final models were verified by visual inspection of the residuals, and no data transformations were required due to the even spread of shark sizes for each species. The alpha level was set at 0.05 for all statistical tests.

## 3 Results

### 3.1 Stomach Content Analysis and Prey Consumption Relative to Body Length

One hundred stomachs of grey nurse sharks (TL range: 95.5–259 cm) and 33 from sevengill sharks (TL range: 69–239 cm) were collected. A total of 92 (92%) stomachs from grey nurse sharks (44 females and 48 males) and 21 (62%) from sevengill sharks (14 females and 7 males) had at least one prey item. Cumulative diversity curves (Figure S1) did not reach an asymptote for either species, indicating that a larger number of stomachs were needed for a more precise description of their diet in this region. Grey nurse sharks preyed mainly on teleosts (92.2%*N*), followed by chondrichthyes (6.7%*N*) (Table 1). Among the most important items were two species of Sciaenidae (striped weakfish, *Cynoscion guatucupa*, and the whitemouth croaker, *Micropogonias furnieri* ) and specimens of the genera *Odontesthes* (silversides). Remains of marine mammals were not found in grey nurse shark stomachs. Based on prey items with low degrees of decomposition, we observed that grey nurse sharks almost always swallowed their prey whole. In comparison, sevengill sharks had a more balanced diet between teleosts (44.7%*N*) and chondrichthyans (34.2%*N*), with some predation on marine mammals (7.9%*N*) (Table 1). No preferred prey item was identified within the defined prey categories, suggesting a more generalized diet. Marine mammals were recorded in three individuals with TL between 146 and 191 cm. Cetaceans occurred in two individuals measuring 146 and 171 cm, while one 191 cm individual fed on a pinniped. Almost all prey items were highly digested; therefore, it was not possible to determine whether prey was eaten whole or in pieces.

**TABLE 1:** Diet composition of *Carcharias taurus* (*n* = 92, grey nurse shark) and *Notorynchus cepedianus* (*n* = 21, sevengill sharks) caught along the Uruguayan coast.

Group/Taxa	Prey	*Carcharias taurus* (*n* = 92)		*Notorynchus cepedianus* (*n* = 21)	
		%*N*	%*F*	%*N*	%*F*
Actinopterygii	—	92.2	94.6	44.7	61.9
Scianidae	*Micropogonias furnieri*	18.7	38.0	5.3	9.5
	*Cynoscion guatucupa*	17.0	35.9	—	—
	*Macrodon atricauda*	4.2	10.9	—	—
	*Paralonchurus brasiliensis*	1.4	4.3	—	—
	*Menticirrhus martinicensis*	0.7	2.2	—	—
	*Umbrina canosai*	0.4	1.1	—	—
Batrachoididae	*Porichthys porosissimus*	9.2	23.9	2.6	4.8
Atherinopsidae	*Odontesthes* sp	17.3	20.7	—	—
Stromateidae	*Peprilus* sp	7.8	13.0	2.6	4.8
Physidae	*Urophycis brasiliensis*	4.6	10.9	—	—
Clupeidae	*Brevoortia aurea*	1.4	3.3	—	—
Trichiuridae	*Trichiurus lepturus*	1.4	3.3	—	—
Mugilidae	*Mugil liza*	1.1	3.3	5.3	9.5
Congridae	*Conger orbignianus*	1.1	3.3	—	—
Sparidae	*Diplodus argenteus*	0.7	2.2	—	—
Ariidae	*Genidens barbus*	—	—	5.3	9.5
Unidentified Teleosts	—	4.6	14.1	23.7	28.6
Chondrichthyes	—	6.7	12.0	34.2	52.4
Subclass – Holocephali	—	0.4	1.1	—	—
Callorhinchidae	*Callorhinchus callorhynchus*	0.4	1.1	—	—
Subclass – Elasmobranchii	—	6.4	10.9	34.2	52.4
Rajidae	*Sympterygia acuta*	0.4	1.1	—	—
	*Sympterygia acuta* (egg capsules)	0.7	1.1	—	—
	*Sympterygia bonapartii*	0.4	1.1	—	—
Myliobatidae	*Myliobatis ridens*	0.7	1.1	5.3	9.5
	*Myliobatis* sp	—	—	2.6	4.8
Squatinidae	*Squatina guggenheim*	0.4	1.1	—	—
Triakidae	*Mustelus schmitti*	1.4	2.2	—	—
Unidentified Batoidea	—	0.4	1.1	7.9	14.3
Unidentified Selachimorpha	—	0.7	2.2	10.5	19.0
Unidentified Elasmobranchs	—	0.4	1.1	7.9	14.3
Unidentified fish species	—	0.7	2.2	2.6	4.8
Marine Mammals	—	—	—	7.9	14.3
Cetacea	Odontocetii	—	—	5.3	9.5
Carnivora	Otariidae	—	—	2.6	4.8

*Note:* Results are presented in percentage by the frequency of occurrence (%*F*) and percentage by number (%*N*).

**FIGURE 3:**
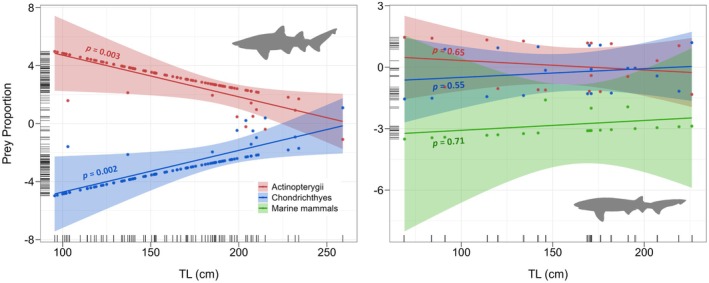
Variation in prey proportion (Logit transformed) as a function of total length (TL) for grey nurse sharks ( *Carcharias taurus* , left panel) and sevengill sharks ( *Notorynchus cepedianus* , right panel). Shaded areas represent 95% confidence intervals. The *p*‐values for each model and the partial residuals are shown, and rugs at the *x* and *y*‐axis represent raw observed data.

Fitted generalised linear models for grey nurse sharks showed significant relationships in the proportion of consumed prey as a function of TL, both for actinopterygii (*p*‐value = 0.003) and chondrichthyes (*p*‐value = 0.002). Decreased consumption of actinopterygii in larger individuals was associated with an increase in consumption of chondrichthyes (Figure 3). No significant relationships were observed between prey proportion as a function of TL for sevengill sharks (actinopterygii *p*‐value = 0.65, chondrichthyes *p*‐value = 0.55, marine mammals *p*‐value = 0.71; Figure 3). No effect of sex on the proportion of consumption of actinopterygii (*p*‐value = 0.27) or chondrichthyes (*p*‐value = 0.27) in relation to TL was found in grey nurse sharks (Table 2, Figure S2). Similar results were observed in sevengill sharks, with no significant effect of sex on the proportion of consumption of actinopterygii (*p*‐value = 0.28) or chondrichthyes (*p*‐value = 0.33) through ontogeny (Table 2, Figure S2).

**TABLE 2:** Differences between sexes in the proportion of types of prey consumed along the total length in grey nurse sharks ( *Carcharias taurus* ) and sevengill sharks ( *Notorynchus cepedianus* ).

	Sex			TL:Sex		
	*z*	*p*	df	*z*	*p*	df
*Carcharias taurus*						
Actinopterygii	1.120	0.26	89	−1.097	0.27	89
Chondrichthyes	−1.120	0.26	89	1.097	0.27	89
*Notorynchus cepedianus*						
Actinopterygii	−1.100	0.27	18	1.079	0.28	18
Chondrichthyes	1.002	0.32	18	−0.968	0.33	18

*Note:* Results of generalized linear models of the proportion of type of prey consumed as a function of total length (TL) and sex as an interaction factor. Prey types analyzed for both species were Actinopterygii and Chondrichthyes. glm(prey proportion)~TL*sex (binomial distribution, logit link function).

Abbreviation: df, degrees of freedom.

### 3.2 Stable Isotope Variation With Body Length

**FIGURE 4:**
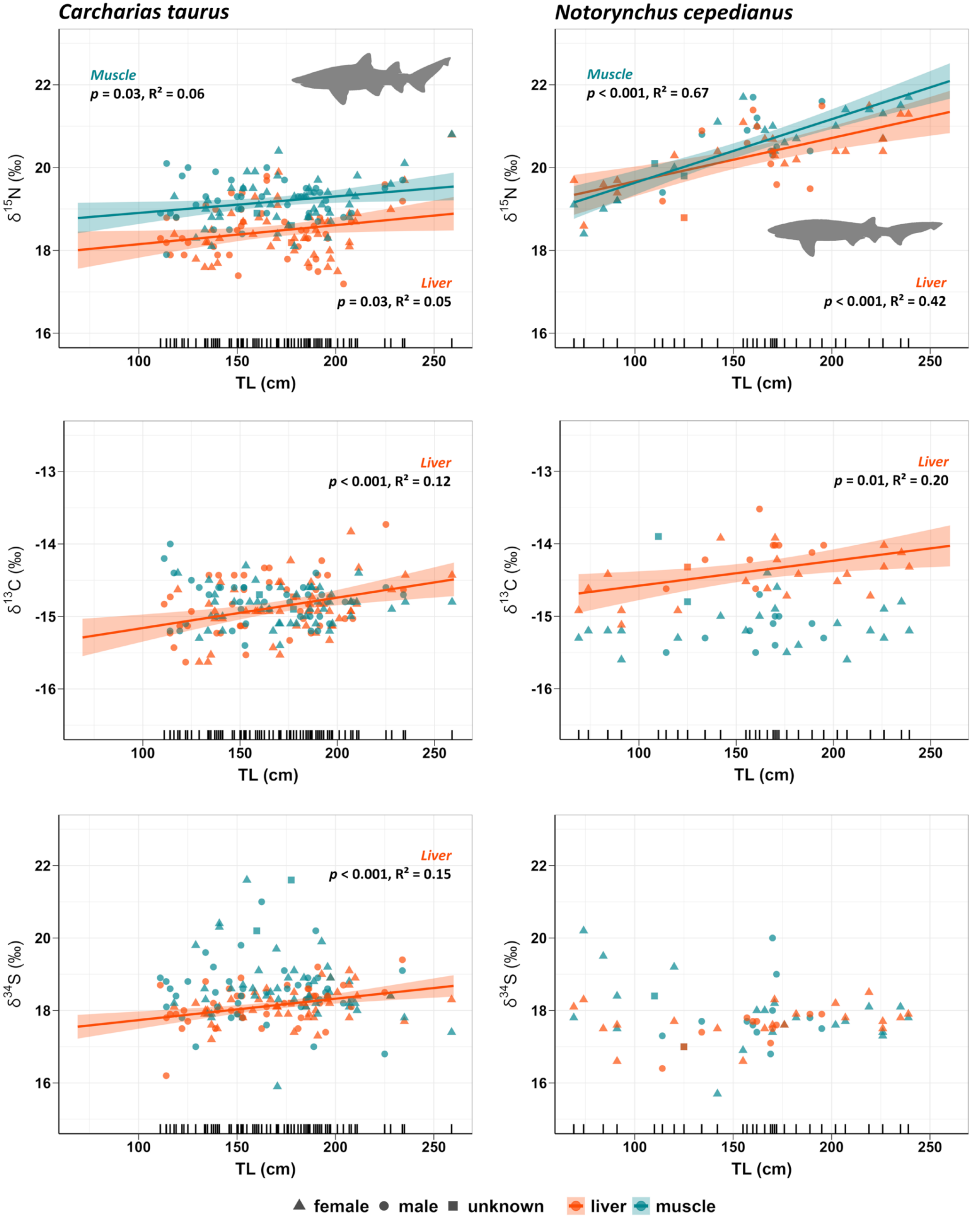
Linear models of δ^15^N, δ^13^C, and δ^34^S values as a function of total length (TL) for each tissue sample, muscle (cyan), and liver (orange) of the grey nurse shark ( *Carcharias taurus* ) and sevengill sharks ( *Notorynchus cepedianus* ). Liver isotopic values for δ^15^N and δ^13^C were adjusted to make them directly comparable to muscle values, see Section 2. Shaded areas represent the 95% confidence intervals. Regression lines are only presented for those variables that showed a significant relationship with total length.

Isotopic values of nitrogen (δ^15^N) were consistently higher in sevengill sharks than in grey nurse sharks, for both tissue types (Table 3). Average values were 1.5‰ and 1‰ higher in liver and muscle, respectively. The range of δ^15^N values varied between 16.5‰ and 20.8‰ in grey nurse sharks and between 17.8‰ and 21.7‰ in sevengill sharks. The range of values for δ^15^N varied slightly in the liver‐corrected values (see Section 2): 17.2‰–20.8‰ in grey nurse sharks and 18.4‰–21.7‰ in sevengill sharks (Figure 4). Sevengill shark showed significant relationships in δ^15^N values as a function of TL, explaining a large proportion of the variation for the muscle (*p*‐value < 0.001, *R* ^2^ = 0.67) and liver (*p*‐value < 0.001, *R* ^2^ = 0.42) (Figure 4). Although the fitted linear models were significant for the muscle and liver in grey nurse sharks, their explained variance was low (muscle: *p*‐value = 0.03, *R* ^2^ = 0.06; liver: *p*‐value = 0.03, *R* ^2^ = 0.05) (Figure 4). Both species showed a positive slope between δ^15^N and TL in the muscle (grey nurse shark 0.004, sevengill shark 0.015) and liver (grey nurse shark 0.005, sevengill shark 0.011), being steeper for both tissue types in sevengill sharks. Comparison of the regression slopes was only significantly different for muscle (muscle *p* < 0.05, liver *p* = 0.07) when comparing both species by the tissue type.

**TABLE 3:** Stable isotope values of nitrogen (δ^15^N), carbon (δ^13^C) and sulphur (δ^34^S) in muscle and liver of grey nurse sharks ( *Carcharias taurus* ) and sevengill sharks ( *Notorynchus cepedianus* ).

	*Carcharias taurus*		*Notorynchus cepedianus*	
	Muscle (*n* = 104)	Liver (*n* = 104)	Muscle (*n* = 34)	Liver (*n* = 31)
δ15_N_	19.2 ± 0.5	17.8 ± 0.6	20.6 ± 0.9	19.5 ± 0.8
δ13_C_	−14.8 ± 0.3	−15.0 ± 0.4	−15.1 ± 0.4	−15.0 ± 0.4
δ34_S_	18.6 ± 0.9	18.1 ± 0.5	17.9 ± 0.9	17.6 ± 0.5

*Note:* Data are expressed as means ± standard deviation and correspond to isotopic values without any adjustment applied to liver samples, see Section 2. Isotopic values are in parts per mill (‰).

Values of the stable isotope of carbon (δ^13^C) from liver increased with TL in both grey nurse (*p* < 0.001, *R* ^2^ = 0.12) and sevengill sharks (*p* = 0.01, *R* ^2^ = 0.20) (Figure 4). However, there was no relationship with TL in muscle of grey nurse (*p* = 0.43, *R* ^2^ = 0.01) and sevengill shark (*p* = 0.89, *R* ^2^ = 0.00) (Figure 4). Carbon isotopic values showed roughly the same values for both species and tissue types and the lowest variance compared with sulphur and nitrogen values (Table 3). Both species varied between −15.8‰ and −13.9‰ in their δ^13^C values. The range of δ^13^C values varied slightly when the liver‐corrected values were considered (see Section 2): between −15.6‰ and −13.7‰ in grey nurse sharks and between −15.6‰ and −13.5‰ in sevengill sharks (Figure 4).

Sulphur stable isotope values (δ^34^S) in muscle had no significant variation in relation to TL in either species (grey nurse shark: *p*‐value = 0.23, *R* ^2^ = 0.02; sevengill shark: *p*‐value = 0.13, *R* ^2^ = 0.07) (Figure 4). In liver samples, there was a significant positive relationship between δ^34^S and TL for grey nurse sharks (*p*‐value < 0.001, *R* ^2^ = 0.15) but not for sevengill sharks (*p*‐value = 0.10, *R* ^2^ = 0.09). Values for δ^34^S varied between 15.9‰ to 21.6‰ in grey nurse sharks and between 15.7‰ to 20.2‰ in sevengill sharks (Figure 4). The level of variance was the same for both species, reaching higher values in muscle samples (Table 3). Differences between species in the mean values were less than 1‰ for both tissues (Table 3).

No significant effect of sex on the variation in stable isotope values (δ^13^C, δ^15^N and δ^3^4S values) relative to TL was detected in either liver or muscle of grey nurse sharks and sevengill sharks (Table 4, Figures S3 and S4). Although the interaction term (TL:Sex) was significant for δ^15^N values in muscle samples of grey nurse sharks (*p*‐value = 0.04), there was only a significant effect of δ^15^N values and TL for females, and no significant relationship was detected in males (see Figure S3).

**TABLE 4:** Linear models evaluating the effect of sex on the stable isotope values as a function of total length (TL).

	*R* ^2^	*F*‐statistic	df	*p*	*t‐*test (sex)	*t*‐test (TL:sex)
*Notorynchus cepedianus*						
d13C~TL*sex (liver)	0.43	6.43	26	**< 0.01**	0.78	0.44
d13C~TL*sex (muscle)	0.07	0.70	28	0.56	0.47	0.52
d15N~TL*sex (liver)	0.43	6.43	26	**< 0.01**	0.80	0.84
d15N~TL*sex (muscle)	0.67	19.09	28	**< 0.01**	0.67	0.75
d34S~TL*sex (liver)	0.24	2.76	26	0.15	**0.04**	0.05
d34S~TL*sex (muscle)	0.12	1.30	28	0.30	0.27	0.27
*Carcharias taurus*						
d13C~TL*sex (liver)	0.06	1.97	98	0.12	0.09	0.08
d13C~TL*sex (muscle)	0.13	4.97	98	**< 0.01**	0.82	0.86
d15N~TL*sex (liver)	0.03	1.04	98	0.38	0.29	0.28
d15N~TL*sex (muscle)	0.17	6.46	98	**< 0.01**	**0.02**	**0.04**
d34S~TL*sex (liver)	0.07	2.62	98	0.06	0.34	0.27
d34S~TL*sex (muscle)	0.07	2.43	98	0.07	0.88	0.97

*Note:* Models were adjusted independently for both types of tissues, liver, and muscle, for grey nurse shark ( *Carcharias taurus* ) and sevengill sharks ( *Notorynchus cepedianus* ), using sex as an interaction term. Significant values are shown in bold format.

Abbreviation: df, degrees of freedom.

## 4 Discussion

Morphological traits associated with feeding activities directly impact the type of prey a marine predator will be able to consume. Teeth specialized in gripping and then swallowing their prey whole with few serrations (e.g., those found in grey nurse sharks), which have evolved several times in vertebrates to facilitate piscivory (Mihalitsis and Bellwood 2021), appear to constrain predators to consuming prey smaller than their gape size (e.g., grey nurse sharks). By contrast, species adapted to cutting their prey into smaller pieces using serrating teeth (e.g., sevengill sharks) can consume prey larger than their mouth gape. We found a significant positive relationship between body length and δ^15^N values, which was steeper in the species capable of sectioning their prey, allowing them to consume higher trophic level prey at smaller sizes. Values of δ^13^C and δ^34^S for both species and tissue types did not show differences of meaningful ecological significance (~1‰), which suggests that both species feed on resources from similar food webs, supporting our contention that the main difference between the two species is the trophic level of prey they consume. Our findings support the hypothesis that the broader prey size selection available to species with cutting teeth may allow them to occupy higher trophic levels (higher δ^15^N values), preying on a wider range of prey sizes than gripping teeth species of similar body size within the same ecosystem.

### 4.1 Morphology and Feeding Ecology

It appears that for these two species, morphological traits directly involved in the feeding process (e.g., teeth) may be drivers of prey choice. As we initially hypothesized, sevengill sharks consumed prey from a broader range of trophic levels, and this was reflected in a wider range and higher δ^15^N values that also increased more rapidly during ontogeny than grey nurse sharks. Shark species with grasping teeth, such as grey nurse sharks must consume individuals smaller than their mouth gape (Mihalitsis and Bellwood 2017), which limits the size of their target prey and restricts their trophic niche. In contrast, predators with teeth that allow them to cut their prey into smaller pieces, such as sevengill sharks and white sharks (Ferrara et al. 2011), can access a wider variety of prey types and sizes, including prey that is larger than their mouth gape. While this study focused on the potential effects of dentition on trophic consumption, there are a number of other morphological factors associated with predation that could explain the patterns observed here. For example, mandibular characteristics such as jaw rigidity and jaw projection are also known to impact prey selection with ontogeny in sharks, with white shark jaw rigidity a key factor in the ontogenetic shift from a diet of fishes to mammals (Ferrara et al. 2011). Moreover, a recent study by López‐Romero et al. (2023) identified specific mandibular characters that are informative for the inferring trophic level and habitat use in Chondrichthyes. Fin morphology is associated with swim speed, efficiency, and maneuverability, which are all associated with capture ability (Norton 1991; Maia et al. 2017; Gayford, Whitehead, and Jaquemet 2024), and these differ substantially in grey nurse sharks and sevengill sharks. However, there is evidence that suggests that sevengill sharks are scavengers rather than active predators, at least for a significant proportion of the marine mammals they consume (see Heithaus 2001). This makes it unlikely that swim performance alone would allow sevengill sharks to scavenge higher trophic level foods like mammals that would not be accessible to grey nurse sharks. Thus, it may be more likely that characteristics associated with prey handling (tooth/jaw morphology and mechanics) that differ between these sharks are driving the observed differences rather than morphological characteristics relating to swimming performance.

Differences in body size, irrespective of gape or tooth morphology, may also be associated with changes in prey choice (Graeb et al. 2006; Lucifora, García, Menni, et al. 2009; Davis et al. 2012) in grey nurse and sevengill sharks. The predator and prey body size are positively correlated in marine environments (Cohen et al. 1993; Costa 2009). Larger body sizes often lead to faster and stronger predators, which facilitate the capture and consumption of larger prey (Wainwright and Richard 1995; Ferrara et al. 2011; Cuthbert et al. 2020), though this sometimes comes at a cost of manoeuvrability and stability (Webb 2005), which can constrain prey selection. Ontogenetic changes in diet occur in many shark species, driven by either changes in body size or due to changes in trophic morphology (McElroy et al. 2006; Baremore et al. 2009; Lucifora, García, Menni, et al. 2009; Dicken et al. 2017). In most fishes, the body size has a positive relationship with mouth size (Karpouzi and Stergiou 2003; Ladds et al. 2020), and a larger gape enables a species to prey on larger prey items from higher trophic levels (Baremore et al. 2009; Powter et al. 2010; Grainger et al. 2020). However, the body size may not always accurately reflect diet choice (Grossnickle 2020; Keppeler et al. 2020), as it is under selective pressures that are not only associated with diet but also with other biological factors, such as reproduction, thermoregulation, and predation risk (Kleiber 1947; Barneche et al. 2018; Tan et al. 2021). For example, from our results, a sevengill shark of 207 cm total length had δ^15^N values of 21.4‰, while a grey nurse shark of the same length had a δ^15^N value of 19.1‰, a difference of about one trophic level. These differences cannot be explained by body size alone, as they are individuals of the same length, and so other characteristics, such as dentition, are more likely to partially explain these patterns. Although the proposed scenario is possibly too simple because it does not consider all possible individual factors involved in prey selection, for example, differences in body mass or behaviour, it highlights the key role of morphology in prey selection and its use in interpreting trophic information. Consequently, for the trophic ecology studies of marine predators, it appears that body size alone is not a sufficient estimator of a predator's diet, and trophic morphological traits such as dental or mandibular characteristics should also be considered.

Sexual dimorphism is common among sharks, affecting traits such as head morphology and dentition (Powter et al. 2010; Berio et al. 2020; Gayford 2023; Gayford, Whitehead, and Jaquemet 2024). In many elasmobranch species exhibiting gyandric heterodonty (sexual dimorphism in dentition), tooth morphology is not solely optimized for feeding; instead, differences are likely related to copulation behavior (e.g., Kajiura and Tricas 1996; Powter et al. 2010; Berio et al. 2020). Additionally, the relationship between body size and other morphological characters can differ between sexes (e.g., Habegger et al. 2012; Gayford, Godfrey, and Whitehead 2023; Gayford, Whitehead, and Jaquemet 2024). Consequently, different relationships between body size, morphology, and trophic dynamics might result in a sex‐biased structure of the dataset. Despite these considerations, we did not detect any sex‐related differences in the relationships between the proportion of prey consumed; however, grey nurse shark females had significantly higher δ^15^N values at larger sizes than males. This could be evidence of gyandric heterodonty, with the teeth or jaw morphology of males more constrained due to copulatory requirements than females. Overall, our results suggest that, for both studied species, sexual dimorphism has at most minor effects on prey selection. However, the sex ratio for sevengill sharks was unbalanced, with approximately twice as many females as males (muscle: 21/11; liver: 20/10; stomachs with prey item; 12/7). Such imbalance, combined with relatively small sample sizes, may limit the detection of potential sex‐related differences. The ability to detect minor effects of sexual dimorphism in the grey nurse sharks in stable isotopes alone highlights the benefits of assessing prey selection using numerous methods.

As expected, both consumers showed an ontogenetic change in prey choice and trophic level. These could be driven by the increased capture performance obtained from growing physically larger (e.g., swim speed/gape) and from slight changes in tooth morphology associated with ontogenetic growth in sharks (Goodman et al. 2022; Soekoe et al. 2022; Powter et al. 2010). Ontogenetic changes in stomach contents were more evident in grey nurse sharks than in sevengill sharks, probably due to the competitive release resulting from increasing gape size. Larger grey nurse sharks consumed a higher proportion of chondrichthyes than smaller sharks, and our findings support earlier research suggesting that this increase is mainly due to an increase in consumption of benthic elasmobranchs (e.g., eagle rays and angel sharks) (Lucifora, García, and Escalante 2009). Lucifora et al. proposed that larger body sizes give the predator the physical strength to handle, kill, and swallow these wide‐bodied prey, which could be difficult and less efficient for a small shark with a smaller gape; this pattern has been observed in bull sharks ( *Carcharhinus leucas* ) where ontogenetic shifts in jaw musculature result in juveniles having access to higher trophic level prey at a comparatively small size (Gayford, Whitehead, and Jaquemet 2024; Habegger et al. 2012). For smaller individuals, feeding on unsuitable prey may lead to substantial costs in terms of handling and processing time, making the prey less attractive (Willson and Hopkins 2011). In sevengill sharks, dietary change throughout their ontogeny was less pronounced probably because smaller individuals have access to prey from higher trophic levels than do grey nurse sharks at similar sizes. In both species, there was a positive relationship between TL and δ^15^N values, confirming that the overall increase in body size as an organism grows allows accessibility to prey at higher trophic levels. However, grey nurse sharks showed a smaller positive slope than sevengill sharks. This was reflected in a smaller variation in the increase of δ^15^N muscle values along with TL, 19.0‰–19.5‰ in grey nurse sharks and 19‰–22‰ in sevengill sharks (Figure 4). A similar ontogenetic increase in δ^15^N values with the body length has also been detected in Australian sevengill shark populations (Abrantes and Barnett 2011). The ontogenetic change in body size increases predator accessibility to larger prey from higher trophic levels; however, the rate at which it increases depends on other morphological traits directly related to feeding activity. Organisms with gripping teeth, without the possibility of severing their prey, show a more moderate increase than those with cutting teeth, as these individuals can access prey from higher trophic levels.

## 5 Conclusions

Trophic morphological traits play an important role in prey selection for predators and may be the primary mechanism in facilitating resource partitioning in sympatric species (DeVries 2017; Figgener et al. 2019; Delariva and Neves 2020). These morphological characteristics result from different evolutionary pathways that result in differential use of resources and strategies to coexist with other species and individuals. However, most morphological traits are under the effect of multiple selective pressures (e.g., feeding, reproduction, thermoregulation, respiration) and distinguishing which of these pressures leads to a morphology is difficult.

In comparative trophic ecology studies with commonly used analyses, such as stable isotopes, fatty acids, or stomach contents, including an assessment of trophic morphological traits may assist in interpreting how extrinsic and intrinsic factors affect the trophic ecology of a predator. We recommend not only focusing on the commonly studied extrinsic factors (inter‐ and intraspecific interactions, and abiotic factors) that might be affecting the trophic ecology of the groups under the study but also on intrinsic factors (morphological and behavioural traits) of the organisms to improve our interpretations.

In future studies, we recommend an evaluation of the effects of other types of tooth morphology on prey selection and trophic niches of predators. This will help to delineate general patterns in marine predators, explaining how these inherent traits constrain or enhance prey selectivity and shape their trophic niches.

## Author Contributions

**Sabrina Riverón:** conceptualization (lead), data curation (lead), formal analysis (lead), funding acquisition (lead), investigation (lead), methodology (lead), project administration (lead), resources (lead), software (lead), validation (lead), visualization (lead), writing – original draft (lead), writing – review and editing (lead). **Vincent Raoult:** conceptualization (equal), data curation (supporting), formal analysis (supporting), funding acquisition (supporting), investigation (supporting), methodology (supporting), project administration (supporting), resources (supporting), software (supporting), supervision (equal), validation (equal), visualization (supporting), writing – original draft (supporting), writing – review and editing (equal). **David J. Slip:** conceptualization (equal), formal analysis (supporting), funding acquisition (supporting), investigation (supporting), methodology (supporting), project administration (supporting), supervision (equal), validation (equal), visualization (supporting), writing – original draft (supporting), writing – review and editing (equal). **Federico Mas:** conceptualization (supporting), data curation (supporting), writing – review and editing (equal). **Martín Laporta:** data curation (supporting), writing – review and editing (equal). **Inés Pereyra:** data curation (supporting), writing – review and editing (equal). **Santiago Silveira:** data curation (supporting), methodology (supporting), writing – review and editing (equal). **Robert G. Harcourt:** conceptualization (equal), formal analysis (supporting), funding acquisition (supporting), investigation (supporting), methodology (supporting), project administration (supporting), resources (supporting), supervision (lead), validation (equal), visualization (supporting), writing – original draft (supporting), writing – review and editing (equal).

## Disclosure

Statement of Inclusion: Our study brings together authors from different countries, including scientists based in the country where the study was conducted. All authors were engaged early on with the research and study design to ensure that their diverse sets and perspectives were considered from the onset. Our research was discussed with local stakeholders to gather feedback on the fieldwork design and collaborate on sample collection after training. Whenever relevant, the literature published by scientists from the region was also cited; efforts were made to consider relevant work published in the local language.

## Conflicts of Interest

The authors declare no conflicts of interest.

## Supporting information


Appendix S1.

## Data Availability

The data that support the findings of this study are subject to legal restrictions due to third‐party agreements. These data were used under licence for this study (Permit number 252/2018) and are managed by the National Directorate of Aquatic Resources (Dirección Nacional de Recursos Acuáticos, DINARA), Ministry of Livestock, Agriculture, and Fisheries (Ministerio de Ganadería, Agricultura y Pesca, MGAP) of Uruguay. As such, restrictions apply to their availability. The raw data have been provided to reviewers for the evaluation process. Access to the data may be granted upon request, subject to approval by the relevant authorities.
