# Supplementary material for: Shearing Tooth Morphology May Allow Sharks to Access Higher Trophic Levels at Smaller Sizes
Source: Ecol Evol. 2025 Jul 29;15(8):e71722. doi: 10.1002/ece3.71722 (PMC12306977; doi:10.1002/ece3.71722)
Supplement: Supplementary file 1 — Appendix S1. [file ECE3-15-e71722-s001.pdf]

SUPPLEMENTARY MATERIAL

Title: Shearing tooth morphology may allow sharks to access higher trophic levels at smaller sizes

Authors: Sabrina Riverón, Vincent Raoult, David J. Slip, Federico Mas, Martín Laporta, Inés Pereyra, Santiago Silveira, Robert G. Harcourt

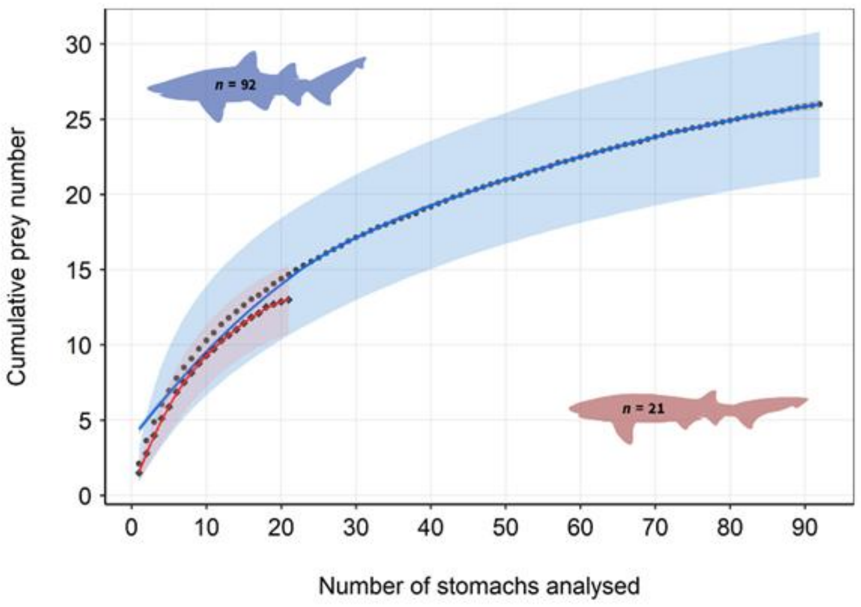

Figure S1. Randomized cumulative prey curves estimated from the stomach content analysis of *Carcharias taurus* (grey nurse shark) and *Notorynchus cepedianus* (sevengill sharks) caught along the Uruguayan coast. The total number of stomachs analyzed with at least one prey item was 92 for grey nurse sharks and 21 for sevengill sharks. The order in which the stomachs were analyzed was randomized 100 times and the means (solid lines) and 95% confidence intervals (shaded area) are plotted.

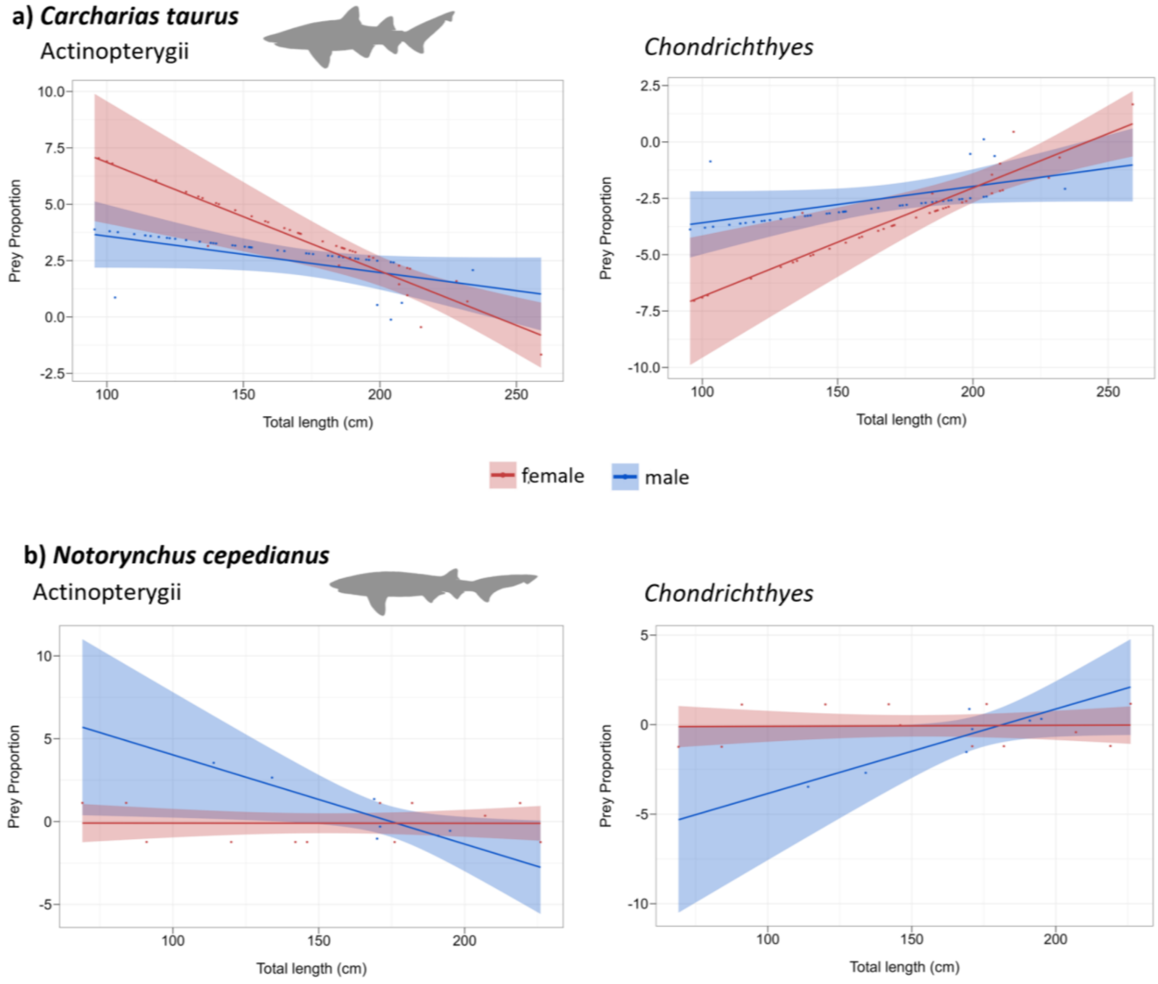

Figure S2. Generalized linear models of Prey Proportion of the grey nurse shark (*Carcharias taurus*) and sevengill sharks (*Notorynchus cepedianus*) as a function of total length (TL) using sex as an interaction. Shaded areas represent the 95% confidence intervals.

Carcharias taurus

Liver **Muscle**

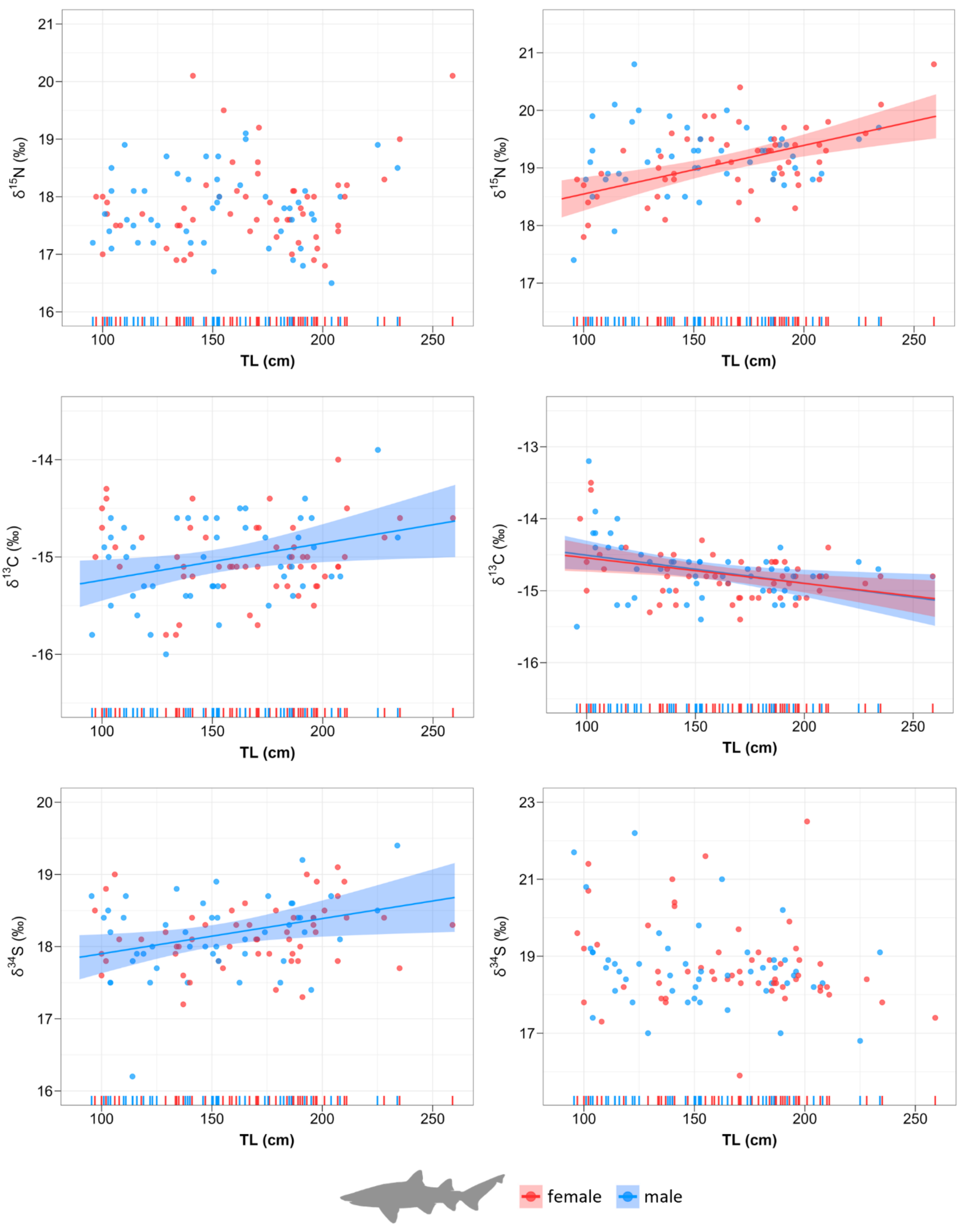

Figure S3. Generalized linear models of $\delta^{15}N$, $\delta^{13}C$ and $\delta^{34}S$ values of the grey nurse shark (*Carcharias taurus*) as a function of total length (TL) for each tissue type (liver and muscle), using sex as an interaction. Shaded areas represent the 95% confidence intervals. Regression lines are only presented for those variables that showed a significant relationship with total length.

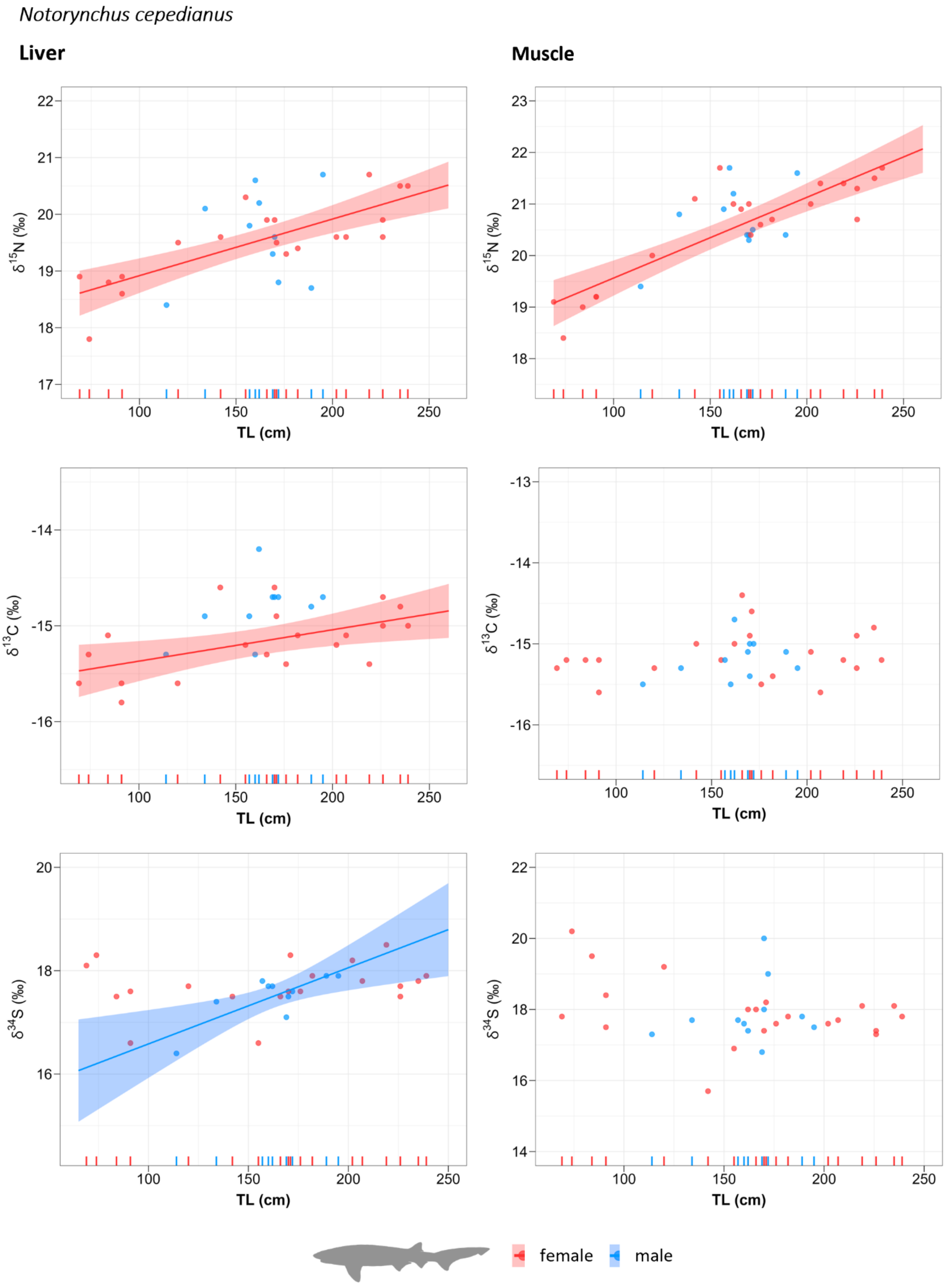

Figure S4. Generalized linear models of $\delta^{15}N$, $\delta^{13}C$ and $\delta^{34}S$ values of the sevengill shark (*Notorynchus cepedianus*) as a function of total length (TL) for each tissue type (liver and muscle), using sex as an interaction. Shaded areas represent the 95% confidence intervals. Regression lines are only presented for those variables that showed a significant relationship with total length.
